# Accuracy and Inter-Subject Variability of Gait Event Detection Methods Based on Optical and Inertial Motion Capture

**DOI:** 10.3390/s25247652

**Published:** 2025-12-17

**Authors:** Vinicius Cavassano Zampier, Morten Bilde Simonsen, Fabio Augusto Barbieri, Anderson Souza Oliveira

**Affiliations:** 1 Human Movement Research Laboratory (MOVI-LAB), Department of Physical Education, School of Sciences, São Paulo State University (UNESP), Bauru 17033-360, Brazil; zampiervc@gmail.com (V.C.Z.);; 2 Department of Materials and Production, Aalborg University, Fibigerstræde 16, Building 4, DK-9220 Aalborg, Denmark

**Keywords:** walking, remote monitoring, gait analysis, validation, IMU

## Abstract

**Highlights:**

Optical motion capture (OMC) methods outperformed inertial measurement units (IMUs) in the accurate detection of gait events. IMU-based methods exhibited higher intra-subject variability to detect heel strike and toe-off events compared with OMC and ground reaction force data.

**What are the main findings?**

- OMC-based methods (OMC1 and OMC2) showed lower estimation errors (RMSE and Range) for heel strike and toe-off detection compared with IMU-based methods (IMU1 and IMU2).
- IMU methods presented a higher intra-subject coefficient of variation (CoV) when used to calculate stance phase durations, indicating greater variability.

**What are the implications of the main findings?**

- OMC remains the preferred choice for applications requiring high temporal accuracy, such as synchronization with EMG or EEG, despite the portability advantages of IMUs.
- The higher intra-subject variability of IMU-based event detection methods may limit their reliability in clinical and longitudinal studies, particularly its usage to segment biological signals.

**Abstract:**

Gait events (instant of heel strikes and instant of toe-offs) are essential for extracting spatiotemporal parameters and segmenting biological signals (electromyography (EMG) and electroencephalography (EEG)) based on gait cycle. While force platforms and optical motion capture (OMC) are ideal for identifying GE, inertial measurement units (IMUs) are more applicable. This study compared the accuracy and variability from IMU- and OMC-based gait event detection methods compared with gold-standard ground reaction force (GRF) detection. Seventeen healthy adults (31 ± 8 years) walked along a 10 m walkway instrumented with force plates. Foot kinematics were recorded using two retro-reflective markers on each foot and an IMU on the sacrum. Gait events were identified using two OMC-based (OMC1, OMC2) and two IMU-based (IMU1, IMU2) algorithms. Accuracy was evaluated using root-mean-square error (RMSE) relative to GRF, and within-subject variability was assessed using coefficient of variation (CoV). The results from the instant of heel strikes, OMC1 yielded a lower RMSE (14 ms) than IMU1 (50 ms) and IMU2 (61 ms) (*p* < 0.001). For the instant of toe-offs, OMC1 demonstrated a lower RMSE (17 ms), differing from IMU1 (54 ms) and IMU2 (74 ms) (*p* < 0.001). IMU2 exhibited greatest variability (CoV = 24 ms) compared with OMC1 (7 ms) and IMU1 (9 ms) (*p* < 0.001). Our results highlight lower accuracy and higher variability in gait event detection using sacrum-mounted IMUs. Despite its convenience, researchers should consider the limitations of using IMUs for EMG/EEG data segmentation. Future studies validating gait event detection methods should report some type of variability metric.

## 1. Introduction

The identification of gait events, such as the instant of heel strikes (i-HS) and the instant of toe-offs (i-TO) during walking, allows the estimation of relevant gait parameters, such as stride, stance and swing durations, double support duration, and gait asymmetry [1,2,3,4,5,6]. The gold-standard method to define gait events involves measurements of ground reaction forces (GRF) [7,8], but force plates are expensive devices that require strict floor installations. One alternative gait event detection method (GEDM) uses optical motion capture (OMC) to detect gait events by tracking the vertical and forward position of markers placed over the calcaneus and the head of the first metatarsus [9]. However, similarly to GRF, OMCs are also not easily accessible for widespread research and clinical applications. Sensor miniaturization has allowed the development of inertial measurement units (IMUs) capable of capturing segment accelerations/rotations during human movements [10,11]. It has been shown that shifts in segment accelerations and rotations can be linked to i-HS and i-TO events [12,13], making inertial motion capture (IMC) technology relevant for gait event detection. Studies use the estimated gait events to calculate gait parameters in healthy people and patient populations, as IMUs are cost-effective, lightweight, and easy to apply [14,15,16]. Both the OMC and IMU methods have been highly relevant to advance our understanding of human motor control and the impact of disorders on mobility, with IMUs in particular allowing recordings in more natural daily life conditions [17,18].

**Figure 1:**
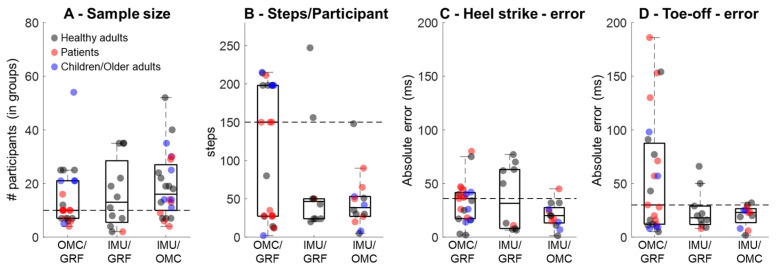
Distribution of sample size (**A**), number of steps per participant (**B**), error in detecting instants of heel strike (**C**), and instants of toe-off (**D**) from optical motion capture validated using ground reaction force (OMC/GRF), from inertial measurement units validated against GRF (IMU/GRF), and from inertial measurement units validated using optical motion capture or pressure sensing systems (IMU/OMC). Horizontal dashed lines represent the median value from OMC/GRF for reference. The study by Wang et al. (2025) [26] investigated 307 healthy older adults and was not included in panel (**A**).

High-quality validations consider sample sizes and the amount of data required for assessment. The literature review on the validation of OMC- and IMU-based GEDMs retrieved a large number of studies, and some of the findings were organized for illustration (Figure 1) and classified as validation of OMC methods against GRF as ground truth [9,19,20,21,22,23,24,25,26] (Supplementary Table S1), validation of IMU-based methods against GRF as ground truth [27,28,29,30,31,32,33,34,35] (Supplementary Table S2), and validation of IMU-based methods using OMC as ground truth [36,37,38,39,40,41,42,43,44,45,46,47,48,49] (Supplementary Table S3). In general, studies recruited 10 to 16 participants (Figure 1A) and used 49 steps/participant for validations of IMU-based methods using OMC to 150 steps/participant for validations of OMC against GRF (Figure 1B). This observation highlights that OMC validations have been performed using a larger number of steps when compared with IMU validations. Regarding accuracy in detecting i-HS, OMC methods validated against GRF present ~36 ms median error, whereas IMU-based methods present lower errors when validated against GRF (~32 ms) or OMCs (~20 ms). Likewise, detections of i-TO from OMC methods validated against GRF present greater median errors (~30 ms) when compared with IMU-based validated against GRF (~18 ms) or OMCs (~23 ms). Moreover, the error when using OMC methods to identify i-HS ranges from 2 ms to 80 ms, reaching up to 200 ms to identify i-TO. Conversely, the errors in studies validating IMU-based methods using OMC are predominantly below 50 ms (Figure 1C,D).

Our exploratory literature review revealed that IMU-based validations yield absolute errors lower than those found when validating OMCs against GRF, two highly accurate and reliable instrumentations. However, this presumed higher accuracy from IMU-based methods can be misleading, as such validations bypass the inherent error from OMC-based GEDMs that are themselves validated against GRF. Surprisingly, no study has made a comprehensive head-to-head comparison of OMC-based and IMU-based GEDMs when both are validated against GRF, highlighting a gap in our current knowledge.

IMU sensor location is also highly relevant for gait event detection. Single IMUs placed on the sacrum have been used in clinical studies [27,37,38,39], as they minimize setup, allow bilateral gait event detection [27,37], and are located in an area that reduces the risk of sensor detachment. However, gait events detected using IMUs placed on the foot or shank are more accurate than those detected from IMUs at other locations [28]. Sacrum-mounted IMUs can be convenient as a supplementary recording during studies investigating bio-signals such as electromyography and/or electroencephalography, allowing segmentation of walking strides and steps [50,51,52]. However, incorrect and/or inconsistent gait event detection might shift the timing of events within the bio-signal, potentially leading to data misinterpretations. Our literature review found only four studies validating sacrum-mounted IMU-based GEDMs against GRF, despite the relevance of this sensor location in clinical applications. Therefore, further assessments of the accuracy of IMU-based methods using a sacrum-mounted sensor are highly relevant and necessary.

Another relevant assessment for validation is the inherent variability of a method when it is applied multiple times. Intra-subject variability (ISV) can be highly relevant in gait event detection, contributing to establishing the robustness of alternative methods. Regarding gait event detections, ISV can demonstrate whether i-HS and i-TO are identified within a narrow error margin across an experimental session. Methods reporting ISV demonstrate superior methodological reliability and reduced measurement error, ultimately supporting the clinical applicability of the technique [53,54]. Furthermore, when assessing gait-related metrics from bio-signals, inconsistent i-HS or i-TO detection might compromise bio-signal segmentation. Interestingly, to the best of our knowledge, there have been no studies validating GEDMs that reported ISV from their validation procedures. Therefore, the aim of this study was to determine the quality of popular IMU- and OMC-based GEDMs against the gold-standard GRFs from the same study sample. Moreover, the ISV from these methods was evaluated based on a large sample of steps. It was hypothesized that OMC-based GEDMs would be more accurate and less variable than IMU-based methods.

## 2. Materials and Methods

### 2.1. Participants

A total of 17 participants (16 male/1 female, age 31 ± 8 years, height 179 ± 6 cm, body mass 78 ± 7 kg) without neurological or musculoskeletal injuries preventing overground walking participated and provided informed consent. All methods followed the Declaration of Helsinki (2004) and were approved by the local Research Ethics Committee (Case number: 2023-505-00139).

### 2.2. Experimental Design and Instrumentation

Participants walked at their preferred speed on an 8 m walkway in a single session. Two force plates (BMS400600, AMTI, Watertown, MA, USA) at the center recorded three-dimensional GRF at 1000 Hz. Participants stepped onto each plate with either foot. Five to seven 60 s trials were recorded per participant while wearing their own footwear. Before recordings, participants were fitted with tri-dimensional IMUs (Trigno Avanti, Delsys Inc., Natick, MA, USA) on the sacrum, recording ±16 g acceleration at 1259 Hz, following the literature recommendations [22,40]. Three-dimensional retro-reflective markers were tracked via a 12-camera OMC (Oqus 300, 310, 500, Qualisys, Göteborg, Sweden, 100 Hz), placed over the calcaneus and first metatarsal. GRF and IMU data were synchronized using QTM software (QTM 2023.2, Qualisys, Göteborg, Sweden, 100 Hz).

### 2.3. Data Processing

i-HS and i-TO events were defined using raw GRF, with a 15 N threshold for both force plates. Calcaneus and metatarsal marker data were interpolated to 1000 Hz and low-pass filtered (4th-order Butterworth, 15 Hz).

Two optical motion capture (OMC) methods were evaluated. The first method (OMC1) [19] consists of identifying major shifts in the vertical velocity profiles of foot markers using a Foot Velocity Algorithm. Specifically, i-HS were defined as the local minima (maximum downward velocity) of the calcaneus marker’s vertical velocity, occurring immediately before ground contact. Conversely, i-TO were identified as the local maxima (maximum upward velocity) of the 1st metatarsal marker’s vertical velocity, which define the onset of the swing phase. The validation study demonstrated that OMC1 was highly accurate [19] and has the advantage of utilizing kinematic data without requiring force thresholds. The second method (OMC2) [9] follows the velocity-threshold approach. Low-pass filtered (4th-order Butterworth, 10 Hz) heel and 1st metatarsal marker trajectories were used to calculate the resultant sagittal velocity, combining vertical and antero-posterior components. Gait events were identified based on the cessation and initiation of foot movement relative to a “quiet” stance phase: (i) i-HS were defined when the heel marker’s sagittal velocity fell below a specific threshold, and (ii) i-TO were defined when the metatarsal marker’s velocity exceeded this threshold. The threshold was dynamically calculated for each step based on the mean plus standard deviation of the marker noise during the stationary contact phase. In our implementation, the window used to calculate these statistics for toe-off detection was increased from the original 50 ms to 75 ms. The modification accounted for low-amplitude movements often present just before the foot leaves the ground, improving detection accuracy for the first metatarsal marker.

Accelerometry data were downsampled to 1000 Hz to match GRF data and were low-pass filtered with a 4th-order Butterworth filter (30 Hz cutoff frequency). Two methods based on distinct signal-processing approaches were used to define gait events. The first method (IMU1) is based on McCamley and co-workers [37], who enhanced the temporal localization of gait events by integrating and differentiating the acceleration signal in three steps. (i) Integration: The vertical acceleration signal (sacrum) first underwent numerical integration using the trapezoidal rule. This step acts as a low-pass filter to attenuate high-frequency noise and enhance fundamental gait features without explicitly estimating velocity or displacement. Therefore, no zero-velocity update or drift correction was applied. (ii) First differentiation (heel-strike detection): The integrated signal was differentiated using a Continuous Wavelet Transform with a Gaussian mother wavelet (e.g., “gaus1”), which acts as a smoothing derivative operator. i-HS were defined as the local minima in this first differentiated signal (S1), corresponding to the sharp changes in acceleration characteristic of initial ground contact. (iii) Second differentiation (toe-off detection): The signal S1 was differentiated a second time, again using the Continuous Wavelet Transform with the same Gaussian basis. i-TO were defined as the local maxima in this second differentiated signal (S2). This decomposition technique effectively localizes cyclical events in non-stationary signals by exploiting the multi-scale nature of the wavelet transform.

The second method (IMU2) is based on the hierarchical algorithm proposed by Sejdić et al. [39]. This method relies on artifact removal and temporal windowing relative to coarse gait features. The processing steps involved the following: (i) Preprocessing: The raw acceleration signals were processed to remove gravity (mean subtraction) and impulse-like artifacts (5th-order median filter), followed by amplitude normalization to reduce inter-subject variability. (ii) Coarse identification: The algorithm first identified “anchor” points for each gait cycle by detecting maximum positive peaks in the vertical acceleration (with a minimum separation of 0.35 s between peaks). (iii) Instant of toe-off detection (Stage 2): i-TO were defined by refining the coarse estimates, specifically by locating the first local minimum (negative peak) in the vertical acceleration, within a 0.15 s window after the identified coarse peak. (iv) Heel-strike detection (Stage 3): i-HS were identified by calculating the absolute first derivative of the antero-posterior signal and locating the minimum value within a 0.15 s window before the corresponding coarse peak (or the largest positive peak in antero-posterior acceleration associated with the step). This multi-stage approach uses the stronger vertical signal to establish the gait-cycle cadence, before refining specific events using the axis most sensitive to each event. The chosen IMU methods were selected for their widespread use in prior literature, as well as their applications in clinical studies, with hundreds of citations at the time of this publication (OMC1: >950 citations, OMC2, IMU1:~200 citations, IMU2: ~40 citations).

An average of 90 ± 19 stance periods per participant was included in the analysis, including GRF data and corresponding periods from OMC1, OMC2, IMU1, and IMU2 for both legs, totaling 1598 steps. No relevant differences were observed between left and right event detections, so results were merged. Root-mean-square error (RMSE) and range of prediction errors were computed by comparing GRF detections with OMC1, OMC2, IMU1, and IMU2. ISV from the estimated stance times and the gait event detection’s RMSE were assessed using the coefficient of variation as illustrated below:


(1) $$ISV=\frac{Standard\;deviation}{mean}\times 100$$

Stance phase time was computed for all methods as the difference between i-HS and the preceding i-TO for the same side.

### 2.4. Statistical Analysis

Statistical analyses were performed using SPSS 20.0 (SPSS, Inc., Chicago, IL, USA), with α = 0.05. Normality and sphericity of dependent variables (stance time, stance time ISV, RMSE, RMSE ISV, and range of prediction errors) were assessed via the Shapiro–Wilk and Mauchly tests, with Greenhouse–Geisser correction when necessary. The effect of gait detection methods on stance time, stance time ISV, and RMSE ISV was assessed using repeated measures one-way ANOVA with Bonferroni post hoc corrections when applicable. Moreover, repeated measures one-way ANOVA was used to verify the accuracy across methods regarding stance RMSE and range of prediction errors, with Bonferroni post hoc correction to explore the differences between the GRF method and the other methods. The effect sizes for the ANOVA results were interpreted using partial eta-squared (η^2^), with thresholds of 0.01, 0.06, and 0.14 indicating small, medium, and large effects, respectively [55]. Pearson correlations assessed stance time agreement between the OMC/IMU methods compared with the GRF method, classified as low (r ≤ 0.3), moderate (0.4 < r ≤ 0.6), or high (r > 0.6) [56]. Bland–Altman plots compared gait event timing, with accuracy defined as mean difference and 95% limits of agreement to assess variability.

## 3. Results

**Figure 2:**
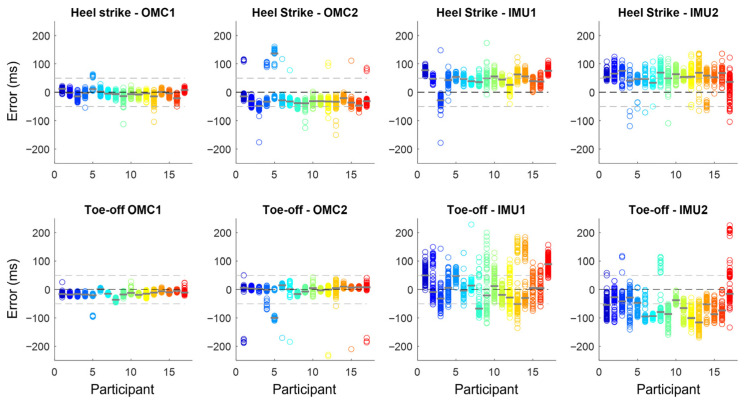
Absolute error (in ms) in instant of heel strike and instant of toe-off identification using two OMC (OMC1 and OMC2) and two IMU methods (IMU1 and IMU2) when compared with the gold-standard identification using ground reaction force. Each circle represents a gait event, and each color represents one of the 17 participants, with the median error represented by the gray line over the circles. Horizontal dashed lines represent no error (0 ms), 50, and −50 ms.

The estimated absolute errors for i-HS and i-TO using the OMC and IMU methods are shown in Figure 2. OMC errors were predominantly within ±50 ms, while IMU methods showed larger errors that reached >200 ms and greater intra-subject dispersion. i-HS were generally detected earlier and i-TO later than actual events, especially for IMUs.

### 3.1. Prediction Quality from OMC and IMU

**Figure 3:**
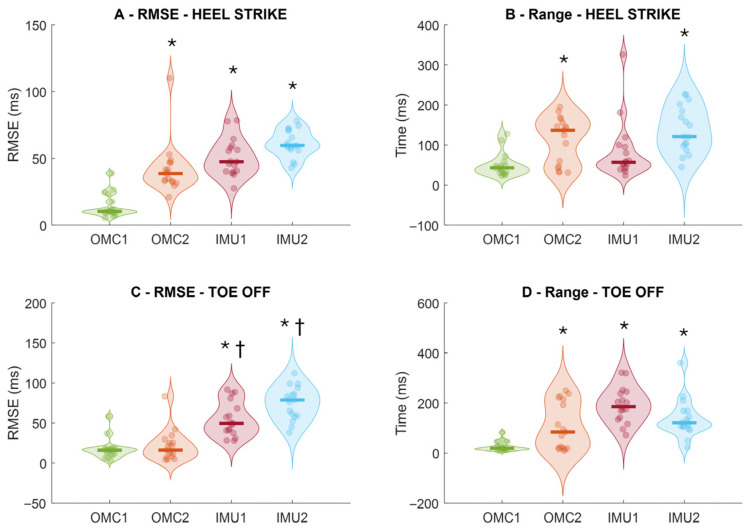
Root-mean-square error (RMSE) of instant of heel strike (**A**) and instant of toe-off (**C**), and intra-subject prediction range of heel strike (**B**) and toe-off (**D**) from the comparison of ground reaction force and methods from optical motion capture (OMC) and inertial measurement units (IMUs) to detect i-HS (upper row) and instant of toe-off events (bottom row). Thick horizontal lines inside the violins represent sample median. * denotes significant different in relation to OMC1 (*p* < 0.01); ^†^ denotes a significant different relation to OMC2 (*p* < 0.05).

There was a significant main effect of method for RMSE for both heel-strike timing (F_3,48_ = 39.079; *p* < 0.001; ηp^2^ = 0.71) and toe-off timing (F_3,48_ = 39.658; *p* < 0.001; ηp^2^ = 0.71). Post hoc comparisons showed that, for heel strikes, OMC1 (14 ± 9 ms) produced significantly lower errors than OMC2 (42 ± 19 ms; *p* < 0.001), IMU1 (50 ± 139 ms; *p* < 0.001), and IMU2 (61 ± 10 ms; *p* < 0.001; Figure 3A). For toe-offs, OMC1 (17 ± 19 ms) also demonstrated significantly lower errors than IMU1 (54 ± 21 ms; *p* < 0.001) and IMU2 (75 ± 20 ms; *p* < 0.001). Additionally, OMC2 (21 ± 19 ms) showed significantly lower toe-off errors than IMU1 (*p* = 0.002) and IMU2 (*p* < 0.001; Figure 3C).

Regarding the range of prediction errors, there was a significant main effect of method for both heel strikes (F_3,48_ = 10.131; *p* < 0.001; ηp^2^ = 0.39) and toe-offs (F_3,48_ = 15.224; *p* < 0.001; ηp^2^ = 0.48). Post hoc analyses revealed that OMC1 (49 ± 29 ms) had significantly lower heel-strike error ranges than OMC2 (112 ± 58 ms; *p* = 0.004) and IMU2 (138 ± 58 ms; *p* < 0.001; Figure 3B). For toe-offs, OMC1 (27 ± 19 ms) showed significantly lower error ranges than OMC2 (107 ± 94 ms; *p* = 0.013), IMU1 (191 ± 70 ms; *p* < 0.001), and IMU2 (138 ± 76 ms; *p* < 0.001; Figure 3D).

**Figure 4:**
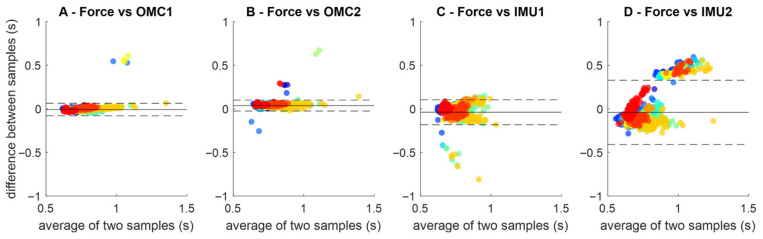
Bland–Altman plots comparing ground reaction force (FORCE) gait event detection to the gait event detection methods OMC1 (**A**), OMC2 (**B**) IMU1 (**C**) and IMU2 (**D**). Each plot contains all stance periods identified across all participants (identified as different colors). The horizontal dashed lines represent the limits of agreement between the stance time and each method.

Bland–Altman plots (Figure 4) showed smaller limits of agreement for OMC1 (0.14 s, Figure 3A) and OMC2 (0.12 s, Figure 3B) than IMU1 (0.28 s, Figure 3C) and IMU2 (0.73 s, Figure 3D). IMU2 stance times displayed a slope, suggesting systematic bias with possible over- or underestimation rather than random variation.

### 3.2. Stance Time Across Methods

**Figure 5:**
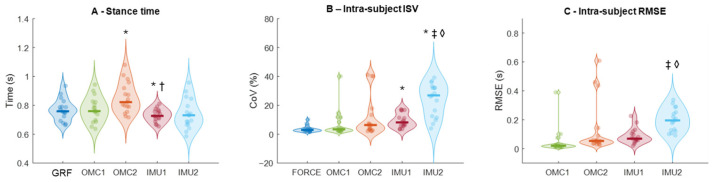
Stance time (**A**), stance time intra-subject variability (ISV, **B**), and stance time intra-subject root-mean-square error (RMSE, **C**) extracted from ground reaction force data and different methods using optical motion capture (OMC) and inertial measurement units (IMU). Thick horizontal lines inside the violins represent sample median. * denotes a significant difference relative to Force (*p* < 0.01); ^†^ denotes a significant difference relative to OMC2 (*p* < 0.05); ^‡^ denotes a significant difference relative to OMC1 (*p* < 0.05). ^◊^ denotes a significant difference relative to OMC (*p* < 0.05).

There was a main effect of method for stance time (F_4,64_ = 12.055; *p* < 0.001; ηp^2^ = 0.43, Figure 5A). Stance time from GRF (0.76 ± 0.01 s) was lower than OMC2 (0.85 ± 0.02 s, *p* = 0.045) and higher than IMU1 (0.73 ± 0.01 s, *p* = 0.045). OMC2 was higher than IMU1 (*p* = 0.001) and IMU2 (0.739 ± 0.025 s, *p* = 0.018). OMC1 overestimated stance by 0.26 ± 5.5%, and OMC2 by 11.4 ± 15.3%. IMU1 underestimated by 4.7 ± 3.8%, and IMU2 by 3.9 ± 8.5%.

### 3.3. Intra-Subject Variability

There was a main effect of method on the ISV calculated from the stance time (F_4,64_ = 12.919; *p* < 0.001; ηp^2^ = 0.44, Figure 5B). ISV was higher in IMU1 (9.2 ± 1.2%, *p* < 0.001) and IMU2 (23.9 ± 2.7%, *p* < 0.001) than in GRF (3.5 ± 0.5%). IMU2 had higher ISV than OMC1 (7.1 ± 2.2%, *p* < 0.001) and IMU1 (*p* = 0.001). GRF measurements presented the lowest CoV (~3.5%). OMC methods averaged 7–12%, while IMU methods averaged 9–24%, with greater inter-subject variability, especially IMU2.

### 3.4. Intra-Subject Root-Mean-Square Error

There was a main effect of method for RMSE (F_3,48_ = 6.105; *p* = 0.010; ηp^2^ = 0.27, Figure 5C). RMSE from OMC1 (0.048 ± 0.02 s) was lower than IMU2 (0.199 ± 0.019 s, *p* < 0.001). RMSE from IMU1 (0.087 ± 0.013 s) was also lower than IMU2 (*p* < 0.001). OMC errors were mostly ≤0.1 s, while IMU errors averaged ~0.15 s, despite some outliers.

### 3.5. Correlation Between Force and OMC/IMU Stance Phase Times

**Figure 6:**
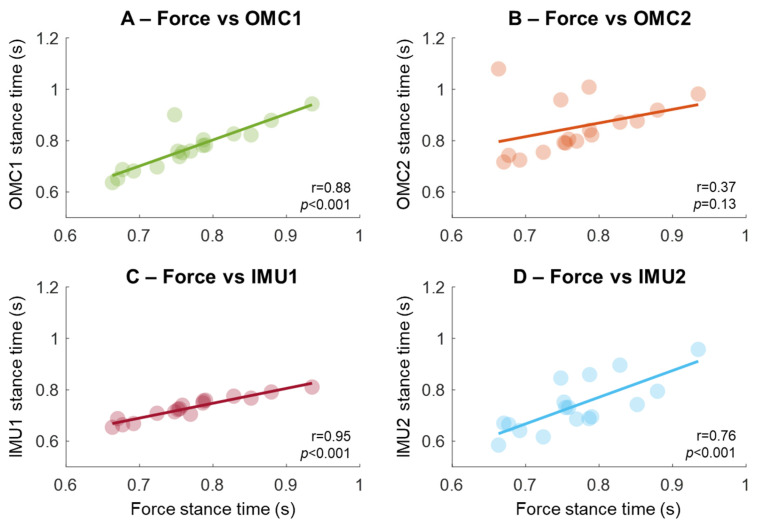
Association of the stance phase time extracted using gold-standard force measurements compared with two methods based on optical motion capture (OMC, **A**,**B**) and two methods using IMU sensors placed on the sacrum (in **C**,**D**). The regression lines were computed from the association of individual participants, represented as circles in the plots.

Stance times from force measurements were highly associated with OMC1 and IMU1 (Figure 6A,C, r > 0.85, *p* < 0.001). Associations were slightly weaker for IMU2 (r = 0.76, *p* < 0.001, Figure 6D), and OMC2 had an outlier reducing correlation to 0.37 (*p* = 0.13, Figure 6B). Removing the outlier increases it to 0.74 (*p* < 0.001).

## 4. Discussion

The main findings of this study were that gait events identified using OMC methods present average errors as low as 15 ms, whereas the best IMU detection reaches approximately 50 ms. Stance time RMSE was lower with OMC1 (48 ± 9 ms) than IMU1 (87 ± 5 ms) and IMU2 (199 ± 5 ms). IMU-based methods also showed greater intra-subject stride-to-stride variability (ISV) for stance time estimates when compared with OMC methods and GRF. Despite discrepancies in variability, there were strong associations between the gold-standard GRF vs. OMC, and between GRF vs. IMU gait event detection methods, when participants are represented by a single average across all detected stance times. Our results suggest that OMC provides more accurate and consistent gait event estimations, while sacrum-mounted IMUs-based methods require caution due to larger errors and greater intra-individual variability. It is recommended that future studies validating GEDMs report intra-subject variability as an additional metric of prediction quality.

### 4.1. Estimation Errors from OMC Methods

Previous studies detecting gait events from OMC methods reported acceptable absolute errors at the range from 4 to 30 ms [22,25,35]. Zeni et al. [19] found average errors of 20–25 ms in young adults, while Caron-Laramée et al. [25] observed similar values when validating OMC-based gait event detection against the gold-standard GRF detection. In Parkinson’s disease, errors reached 30 ms [50]. The OMC algorithms tested in this study had an average error of ~25 ms, corroborating the errors reported in previous studies [19]. Moreover, OMC methods showed lower ISV (~7–12%) than IMU-based methods (~9–24%), suggesting more consistent detection errors and superior performance of OMC-based methods in estimating i-HS and i-TO. Bonci et al. [57] performed a comprehensive comparison of studies validating OMC-based methods, such as those by Zeni et al. [19] and Ghoussayni et al. [9]. These cited studies were conducted with few healthy participants and a limited number of steps, potentially limiting robustness and generalizability of their outcomes. In contrast, our study analyzed 16 participants and ~1600 overground steps (~90 steps/participant) using synchronized force plates, OMC, and IMUs, providing a more comprehensive and ecologically valid benchmark.

### 4.2. Prediction Errors from IMU Methods

The literature on sacrum-mounted IMU-based GEDMs shows their feasibility in estimating gait parameters by detecting i-HS and i-TO but also highlights inconsistencies in validations against gold-standard GRF detection. Bugané et al. [33] compared ~220 steps obtained with a sacrum-mounted IMU method to gold-standard GRF detection, reporting high accuracy for step length and duration, but not reporting temporal differences for gait events. González et al. [34] found errors of ~13 ms (i-HS) and 9 ms (i-TO) when validating against GRF, but data were acquired from only six participants and 222 steps in total. A study with larger sample size [49] reported errors up to 151 ms (i-HS) and 139 ms (i-TO) across 504 steps from ten participants. However, the study was conducted using treadmill walking, limiting the generalizability to overground conditions. This gap underscores the contribution of the present study, in which IMU-based GEDMs were tested and their accuracies were evaluated against GRF using a larger dataset. Our results revealed lower accuracy (errors > 50 ms) and greater intra-subject variability (>15%) for established IMU-based methods compared with OMC-based methods and the gold-standard GRF detection.

The lower prediction quality of sacrum-mounted IMUs with reference to GRF may relate to a temporal mismatch between actual gait events and detectable patterns in the IMU data. Peaks/valleys within the IMU data may indicate foot contact, but they may not be fully aligned to touch-down or toe-off due to mechanical propagation delays. This likely causes the systematic pattern in our data, where i-HS are detected earlier and i-TO later, especially for the method IMU2, reflecting bias from sacrum location and detection logic. While these biases reduce event-level temporal accuracy, they could potentially be corrected or considered when interpreting time-dependent physiological signals. Signal transformations (e.g., wavelets) may improve event detection but still leave events temporally misaligned. Despite the temporal mismatches, inter-subject stance time patterns remained unaffected, as shown by strong associations between prediction methods (OMC/IMU) and the gold-standard GRF detection. It is noteworthy that these strong associations were achieved using ~90 steps to generate an average value for each participant. Previous studies have demonstrated that data averaging can substantially improve robustness [38], potentially masking possible flaws in the event detection process. Therefore, our study also contributes to the field by shedding light on possible limitations on the use of average data to represent individuals in studies dedicated to validating GEDMs.

Our results demonstrate that the IMU-based methods presented greater ISV (IMU1: ~9.2%; IMU2: ~23.9%) than OMC methods (OMC1: ~7.1%; OMC2: ~12%) and the GRF method (3.5%) when stance times were estimated. To our knowledge, this is the only study evaluating the ISV or step-to-step variability of GEDMs during overground walking. Previous studies have evaluated intraclass correlation coefficients (ICCs) to describe reliability and validity of proposed methods such as IMU1, reporting acceptable reliability for step time, step length, and step velocity [38,40], despite the high ISV observed for individual gait event detections. It is common practice in the literature to average i-HS and i-TO across several steps to report individual data, which may dilute individual event detection errors through an averaging effect [16,58]. For instance, Aminian et al. [58] showed that systematic delays of ~10 ms in individual IMU-based event detection did not influence significantly the method’s accuracy when calculating spatiotemporal parameters. This error dilution phenomenon implies that gait parameters derived from highly variable i-HS and i-TO events can still demonstrate acceptable reliability for clinical applications, despite high ISV. However, this high ISV in identifying gait events may compromise applications requiring precise temporal alignment, such as EMG/EEG signal segmentation.

It is noteworthy that OMC-based GEDMs have been considered the “ground truth” in previous studies validating IMC-based methods. However, previous studies and our results demonstrate that some OMC-based methods may introduce considerably greater errors compared with GRF detection, as revealed by the results from the OMC2 method in this study. Therefore, researchers and clinicians interested in assessing gait events should prioritize the use of GRF whenever possible. Likewise, future research validating IMU-based GEDMs should prioritize the use of GRF as ground truth to maximize validation quality, since surprisingly few studies have used GRF for this purpose.

### 4.3. Limitations

This study has some limitations. Firstly, only GEDMs based on sacrum-mounted IMUs were investigated, although placing IMUs on the shanks and feet may yield more accurate gait event detection [43]. However, the sacrum-mounted IMU location was intentionally prioritized because it is highly relevant in clinical research [40,59,60], as fitting a single sensor on the sacrum reduces setup time allows bilateral gait event detection and does not interfere with natural walking. Nevertheless, having similar analysis from sensors placed on the shank or feet would have enabled a clearer understanding of the sources of error, variability, and bias observed in sacral IMU signals. Therefore, the present findings should primarily be interpreted in the context of sacrum-mounted IMUs during overground walking and may not fully generalize to configurations using shank- or foot-mounted sensors, or to other locomotor tasks. Future studies should incorporate multiple IMU locations, including foot or shank positions, to evaluate the effect of sensor location on event detection accuracy.

Secondly, the present study did not implement recent and more sophisticated gait event detection methods based on machine learning applied to sacrum-mounted IMU data. Such studies have shown errors below 14 ms for i-HS and 21 ms for i-TO [26,61] from large datasets. These approaches may outperform the rule-based IMU algorithms examined here, and their relative ranking compared with OMC- and GRF-based methods might differ from the patterns observed in our study. However, machine learning-based GEDMs typically require large datasets, dedicated training/validation pipelines, and task-specific tuning, which were beyond the scope of the present methodological comparison. Future studies assessing the intra-subject variability and accuracy of machine learning-based GEDMs across different sensor locations and populations are therefore highly relevant.

Finally, our study sample consisted predominantly of male participants and therefore may not capture potential sex-related factors in gait event detection. Previous studies have shown that older females exhibit reduced minimum toe clearance compared with males [62], as well as sex-related differences in stride length and walking speed, despite no differences between males and females for heel-strike detection [63]. As a result, the generalization of our findings to female populations, particularly in older adults, should be made with caution. These results support the need for future investigations explicitly designed with sex-balanced samples and sufficient power to compare gait event detection performance between males and females.

## 5. Conclusions

In summary, OMC-based GEDMs showed higher accuracy and lower intra-subject variability than IMU-based methods, especially OMC1. Despite the lower accuracies and higher variability found for IMU-based methods, stance times estimated from IMU-based gait events were similar to those estimated from the OMC or GRF methods. Similarly, the association between stance times estimated using IMUs and GRF was as strong as that between stance times from OMC- and GRF-based methods. Such comparable quality highlights the strong effect of data averaging when representing individual spatiotemporal gait parameters.

In practice, OMC- and IMU-based GEDMs offer complementary advantages depending on the application context. IMUs provide a practical, cost-effective solution for real-world gait monitoring if their limitations—especially lower accuracy and higher intra-subject variability—are acknowledged in data interpretation. However, researchers extracting i-HS and i-TO from IMU-based methods to segment gait cycles must be cautious regarding the inherent variability of these methods, as erroneous gait segmentation can negatively influence their analyses (e.g., EMG, EEG measurements).

Our study highlights the need for rigorous assessment of alternative GEDMs using sufficient sample sizes and numbers of steps/events for validation. Moreover, future studies validating GEDMs—from OMC-, IMU-, or pressure-based systems—should report some type of intra-subject variability metric.

## Data Availability

Data will be available when requested.

## Supplementary Materials

The following supporting information can be downloaded at https://www.mdpi.com/article/10.3390/s25247652/s1, Table S1: Studies validating gait event detection methods based on optical motion capture against ground reaction force detection; Table S2: Studies validating gait event detection methods based on inertial measurement units (IMUS) against ground reaction force detection; Table S3: Studies validating gait event detection methods based on inertial measurement units (IMUs) against optical motion capture (OMC), and pressure-based techniques (foot switches, pressure mats and insoles.

## Abbreviations

The following abbreviations are used in this manuscript:

GRF	Ground Reaction Forces
GEDM	Gait event detection method
ISV	Intra-Subject variability
IMUs	Inertial Measurement Units
OMC	Optical Motion Capture
EMG	Electromyography
EEG	Electroencephalography
RMSE	Root-mean-square error
CoV	Coefficient of Variation

## References

[1] Gaßner H., Sanders P., Dietrich A., Marxreiter F., Eskofier B.M., Winkler J., Klucken J. (2020). Clinical relevance of standardized mobile gait tests. Reliability analysis between gait recordings at hospital and home in Parkinson’s disease: A pilot study. J. Park. Dis..

[2] Tabard-Fougère A., Rutz D., Pouliot-Laforte A., De Coulon G., Newman C.J., Armand S., Wegrzyk J. (2022). Are clinical impairments related to kinematic gait variability in children and young adults with cerebral palsy?. Front. Hum. Neurosci..

[3] Ditunno J., Scivoletto G. (2009). Clinical relevance of gait research applied to clinical trials in spinal cord injury. Brain Res. Bull..

[4] Faria M.H., Simieli L., Rietdyk S., Penedo T., Santinelli F.B., Barbieri F.A. (2023). (A) symmetry during gait initiation in people with Parkinson’s disease: A motor and cortical activity exploratory study. Front. Aging Neurosci..

[5] Godi M., Arcolin I., Giardini M., Corna S., Schieppati M. (2021). A pathophysiological model of gait captures the details of the impairment of pace/rhythm, variability and asymmetry in Parkinsonian patients at distinct stages of the disease. Sci. Rep..

[6] Vitorio R., Mancini M., Carlson-Kuhta P., Horak F.B., Shah V.V. (2023). Should we use both clinical and mobility measures to identify fallers in Parkinson’s disease?. Park. Relat. Dis..

[7] Marasović T., Cecić M.O.J.M.I.L., Zanchi V. (2009). Analysis and interpretation of ground reaction forces in normal gait. WSEAS Trans. Syst..

[8] Samson M.M., Crowe A., De Vreede P.L., Dessens J.A., Duursma S.A., Verhaar H.J. (2001). Differences in gait parameters at a preferred walking speed in healthy subjects due to age, height and body weight. Aging Clin. Exp. Res..

[9] Ghoussayni S., Stevens C., Durham S., Ewins D. (2004). Assessment and validation of a simple automated method for the detection of gait events and intervals. Gait Posture.

[10] Hindle B.R., Keogh J.W., Lorimer A.V. (2021). Inertial-Based Human Motion Capture: A Technical Summary of Current Processing Methodologies for Spatiotemporal and Kinematic Measures. Appl. Bionics Biomech..

[11] Staab W., Hottowitz R., Sohns C., Sohns J.M., Gilbert F., Menke J., Lotz J. (2014). Accelerometer and gyroscope based gait analysis using spectral analysis of patients with osteoarthritis of the knee. J. Phys. Ther. Sci..

[12] Micó-Amigo M.E., Bonci T., Paraschiv-Ionescu A., Ullrich M., Kirk C., Soltani A., Küderle A., Gazit E., Salis F., Alcock L. (2023). Assessing real-world gait with digital technology? Validation, insights and recommendations from the Mobilise-D consortium. J. Neuroeng. Rehabil..

[13] Rochester L., Mazzà C., Mueller A., Caulfield B., McCarthy M., Becker C., Miller R., Piraino P., Viceconti M., Dartee W.P. (2020). A Roadmap to Inform Development, Validation and Approval of Digital Mobility Outcomes: The Mobilise-D Approach. Digit. Biomark..

[14] Del Din S., Hickey A., Ladha C., Stuart S., Bourke A.K., Esser P., Godfrey A. (2016). Instrumented gait assessment with a single wearable: An introductory tutorial. F1000Research.

[15] de Oliveira Gondim I.T.G., de Souza C.D.C.B., Rodrigues M.A.B., Azevedo I.M., de Sales M.D.G.W., Lins O.G. (2020). Portable accelerometers for the evaluation of spatio-temporal gait parameters in people with Parkinson’s disease: An integrative review. Arch. Gerontol. Geriatr..

[16] He Y., Chen Y., Tang L., Chen J., Tang J., Yang X., Xiao N. (2024). Accuracy validation of a wearable IMU-based gait analysis in healthy female. BMC Sports Sci. Med. Rehabil..

[17] Wagenaar R.C., van Emmerik R.E. (2000). Resonant frequencies of arms and legs identify different walking patterns. J. Biomech..

[18] Zampier V.C., Mochizuki L., Beretta V.S., de Belli V., Gobbi L.T.B., Barbieri F.A., Orcioli-Silva D. (2025). Verbal arm swing instructions alter arm-leg interlimb coordination but not prefrontal cortex hemodynamics in people with Parkinson’s disease. Hum. Mov. Sci..

[19] Zeni J.A., Richards J.G., Higginson J. (2008). Two simple methods for determining gait events during treadmill and overground walking using kinematic data. Gait Posture.

[20] O’Connor C.M., Thorpe S.K., O’Malley M.J., Vaughan C.L. (2007). Automatic detection of gait events using kinematic data. Gait Posture.

[21] Lambrecht S., Harutyunyan A., Tanghe K., Afschrift M., De Schutter J., Jonkers I. (2017). Real-time gait event detection based on kinematic data coupled to a biomechanical model. Sensors.

[22] Zahradka N., Verma K., Behboodi A., Bodt B., Wright H., Lee S.C. (2020). An evaluation of three kinematic methods for gait event detection compared to the kinetic based ‘gold standard’. Sensors.

[23] French M.A., Koller C., Arch E.S. (2020). Comparison of three kinematic gait event detection methods during overground and treadmill walking for individuals post stroke. J. Biomech..

[24] Gómez-Pérez C., Martori J.C., Diví A.P., Casanovas J.M., Samsó J.V., Font-Llagunes J.M. (2021). Gait event detection using kinematic data in children with bilateral spastic cerebral palsy. Clin. Biomech..

[25] Caron-Laramée A., Walha R., Boissy P., Gaudreault N., Zelovic N., Lebel K. (2023). Comparison of three motion capture-based algorithms for spatiotemporal gait characteristics: How do algorithms affect accuracy and precision of clinical outcomes?. Sensors.

[26] Wang S., Omar K.S., Miranda F., Bhatt T. (2025). Automatic gait EVENT detection in older adults during perturbed walking. J. Neuroeng. Rehabil..

[27] Zijlstra W., Hof A.L. (2003). Assessment of spatio-temporal gait parameters from trunk accelerations during human walking. Gait Posture.

[28] Boutaayamou M., Schwartz C., Stamatakis J., Denoël V., Maquet D., Forthomme B., Brüls O. (2015). Development and validation of an accelerometer-based method for quantifying gait events. Med. Eng. Phys..

[29] Maqbool H.F., Husman M.A.B., Awad M.I., Abouhossein A., Iqbal N., Dehghani-Sanij A.A. (2016). A real-time gait event detection for lower limb prosthesis control and evaluation. IEEE Trans. Neural Syst. Rehabil. Eng..

[30] Panebianco G.P., Bisi M.C., Stagni R., Fantozzi S. (2018). Analysis of the performance of 17 algorithms from a systematic review: Influence of sensor position, analysed variable and computational approach in gait timing estimation from IMU measurements. Gait Posture.

[31] Strick J.A., Farris R.J., Sawicki J.T. (2024). A novel gait event detection algorithm using a thigh-worn inertial measurement unit and joint angle information. J. Biomech. Eng..

[32] Yang S., Koo B., Lee S., Jang D.J., Shin H., Choi H.J., Kim Y. (2024). Determination of gait events and temporal gait parameters for persons with a knee–ankle–foot orthosis. Sensors.

[33] Bugané F., Benedetti M.G., Casadio G., Attala S., Biagi F., Manca M., Leardini A. (2012). Estimation of spatial-temporal gait parameters in level walking based on a single accelerometer: Validation on normal subjects by standard gait analysis. Comput. Methods Programs Biomed..

[34] González R.C., López A.M., Rodríguez-Uría J., Álvarez D., Álvarez J.C. (2010). Real-time gait event detection for normal subjects from lower trunk accelerations. Gait Posture.

[35] Hansen A.H., Childress D.S., Meier M.R. (2002). A simple method for determination of gait events. J. Biomech..

[36] Jasiewicz J.M., Allum J.H., Middleton J.W., Barriskill A., Condie P., Purcell B., Li R.C.T. (2006). Gait event detection using linear accelerometers or angular velocity transducers in able-bodied and spinal-cord injured individuals. Gait Posture.

[37] McCamley J., Donati M., Grimpampi E., Mazzà C. (2012). An enhanced estimate of initial contact and final contact instants of time using lower trunk inertial sensor data. Gait Posture.

[38] Godfrey A., Del Din S., Barry G., Mathers J.C., Rochester L. Within trial validation and reliability of a single tri-axial accelerometer for gait assessment. Proceedings of the 36th Annual International Conference of the IEEE Engineering in Medicine and Biology Society.

[39] Sejdić E., Lowry K.A., Bellanca J., Perera S., Redfern M.S., Brach J.S. (2015). Extraction of stride events from gait accelerometry during treadmill walking. IEEE J. Transl. Eng. Health Med..

[40] Del Din S., Godfrey A., Rochester L. (2015). Validation of an accelerometer to quantify a comprehensive battery of gait characteristics in healthy older adults and Parkinson’s disease: Toward clinical and at-home use. IEEE J. Biomed. Health Inform..

[41] Romijnders R., Warmerdam E., Hansen C., Welzel J., Schmidt G., Maetzler W. (2021). Validation of IMU-based gait event detection during curved walking and turning in older adults and Parkinson’s Disease patients. J. Neuroeng. Rehabil..

[42] Nazarahari M., Khandan A., Khan A., Rouhani H. (2022). Foot angular kinematics measured with inertial measurement units: A reliable criterion for real-time gait event detection. J. Biomech..

[43] Voisard C., de l’Escalopier N., Ricard D., Oudre L. (2024). Automatic gait events detection with inertial measurement units: Healthy subjects and moderate to severe impaired patients. J. Neuroeng. Rehabil..

[44] Brahimetaj R., Zaccardi S., Bautmans I., Jansen B. (2024). Assessing two IMU-based gait event detection methods and their effect on spatiotemporal gait parameters across young and elderly populations. Gait Posture.

[45] Digo E., Panero E., Agostini V., Gastaldi L. (2023). Comparison of IMU set-ups for the estimation of gait spatio-temporal parameters in an elderly population. Proc. Inst. Mech. Eng. Part H J. Eng. Med..

[46] Hundza S.R., Hook W.R., Harris C.R., Mahajan S.V., Leslie P.A., Spani C.A., Livingston N.J. (2013). Accurate and reliable gait cycle detection in Parkinson’s disease. IEEE Trans. Neural Syst. Rehabil. Eng..

[47] Kluge F., Gaßner H., Hannink J., Pasluosta C., Klucken J., Eskofier B.M. (2017). Towards mobile gait analysis: Concurrent validity and test-retest reliability of an inertial measurement system for the assessment of spatio-temporal gait parameters. Sensors.

[48] Larsen A.G., Sadolin L.Ø., Thomsen T.R., Oliveira A.S. (2024). Accurate detection of gait events using neural networks and IMU data mimicking real-world smartphone usage. CMBBE.

[49] Lee J.A., Cho S.H., Lee Y.J., Yang H.K., Lee J.W. (2010). Portable activity monitoring system for temporal parameters of gait cycles. J. Med. Syst..

[50] Weersink J.B., Maurits N.M., de Jong B.M. (2019). EEG time-frequency analysis provides arguments for arm swing support in human gait control. Gait Posture.

[51] Weersink J.B., Maurits N.M., de Jong B.M. (2021). Amble gait EEG points at complementary cortical networks underlying stereotypic multi-limb co-ordination. Front. Hum. Neurosci..

[52] Agostini V., Ghislieri M., Rosati S., Balestra G., Knaflitz M. (2020). Surface electromyography applied to gait analysis: How to improve its impact in clinics?. Front. Neurol..

[53] Monoli C., Galli M., Tuhtan J.A. (2024). Improving the reliability of underwater gait analysis using wearable pressure and inertial sensors. PLoS ONE.

[54] Rinderknecht M.D., Lambercy O., Gassert R. (2018). Enhancing simulations with intra-subject variability for improved psychophysical assessments. PLoS ONE.

[55] Richardson J.T. (2011). Eta squared and partial eta squared as measures of effect size in educational research. Educ. Res. Rev..

[56] Akoglu H. (2018). User’s guide to correlation coefficients. Turk. J. Emerg. Med..

[57] Bonci T., Salis F., Scott K., Alcock L., Becker C., Bertuletti S., Mazzà C. (2022). An algorithm for accurate mark-er-based gait event detection in healthy and pathological populations during complex motor tasks. Front. Bioen-Gineering Biotechnol..

[58] Aminian K., Najafi B., Büla C., Leyvraz P.F., Robert P. (2002). Spatio-temporal parameters of gait measured by an ambulatory system using miniature gyroscopes. J. Biomech..

[59] Elshehabi M., Del Din S., Hobert M.A., Warmerdam E., Sünkel U., Schmitz-Hübsch T., Behncke L.M., Heinzel S., Brockmann K., Metzger F.G. (2022). Walking parameters of older adults from a lower back inertial measurement unit, a 6-year longitudinal observational study. Front. Aging Neurosci..

[60] Godfrey A., Del Din S., Barry G., Mathers J.C., Rochester L. (2015). Instrumenting gait with an accelerometer: A system and algorithm examination. Med. Eng. Phys..

[61] Arshad M.Z., Jamsrandorj A., Kim J., Mun K.R. (2022). Gait Events Prediction Using Hybrid CNN-RNN-Based Deep Learning Models through a Single Waist-Worn Wearable Sensor. Sensors.

[62] Faria A., Sousa T., Vaz J.R., Gabriel R., Gama J., Stergiou N. (2024). Females Present Reduced Minimum Toe Clearance During Walking As Compared to Males in Active Older Adults. J. Gerontol. A Biol. Sci. Med. Sci..

[63] Al-Makhalas A., Abualait T., Ahsan M., Abdulaziz S., Al Muslem W. (2023). A gender based comparison and correlation of spatiotemporal gait parameters and postural stability. Acta Biomed..

