# Supplementary material for: Accuracy and Inter-Subject Variability of Gait Event Detection Methods Based on Optical and Inertial Motion Capture
Source: Sensors (Basel). 2025 Dec 17;25(24):7652. doi: 10.3390/s25247652 (PMC12736683; doi:10.3390/s25247652)
Supplement: Supplementary file 1 [file sensors-25-07652-s001.zip › sensors-4000881-supplementary.pdf]

Supplementary Table 1. Studies validating gait event detection methods based on optical motion capture against ground reaction force detection. **OVG**: OVG; **TRD**: TRD; **nr**: not recorded.

Author (year)	Population	Sample size	Floor	Steps	TotalSteps	Error HS (ms)	Error TO (ms)
**Ghoussayni (2004) [9]**	Healthy adults	12	Overgroud	12	144	16	16
**Oconnor (2007) [20]**	Healthy children	54	OVG	2	126	16	9
**Zeni (2008) [19]**	Healthy adults	7	TRD	27	191	17	11
	Multiple sclerosis	7	TRD	35	247	37	12
	Stroke	4	TRD	27	110	26	16
	Healthy adults	7	OVG	14	102	2	12
**Lambrecht (2017) [21]**	Healthy adults	7	TRD	80	558	3	43
**Zahradka (2020) [22]**	Healthy adults	6	TRD	214	1502	40	5
	Healthy children	5	TRD	215	1506	42	8
	Children cerebral palsy	6	TRD	211	1480	80	20
**French (2020) [23]**	Adults with Stroke	10	OVG	28	284	44	30
	Adults with Stroke	10	OVG	28	284	18	130
	Adults with Stroke	10	OVG	28	284	39	57
	Adults with Stroke	10	TRD	150	1502	46	28
	Adults with Stroke	10	TRD	150	1502	36	186
	Adults with Stroke	10	TRD	150	1502	38	71
**Gómez-Pérez (2021) [24]**	Children cerebral palsy	16	OVG	12	202	nr	nr
**Caron-Laramee (2023) [25]**	Healthy adults - Slow walking	25	TRD	198	9114	47	153
	Healthy adults - Usual walking	25	TRD	198	9114	34	91
	Healthy adults - Fast walking	25	TRD	198	9114	25	77
	Elderly - Slow walking	21	TRD	198	9114	75	154
	Elderly - Usual walking	21	TRD	198	9114	26	98
	Elderly - Fast walking	21	TRD	198	9114	40	57
**Wang (2025) [26]**	Healthy older adults	307	OVG	nr	nr	14	10
**Median**		**8.5**		**28**	**284**	**36**	**30**
**Mean**		**34.79**		**80.1**	**1146.67**	**33.1**	**56.26**
**SD**		**79.45**		**84.8**	**2081**	**19.3**	**54.9**

Supplementary Table 2. Studies validating gait event detection methods based on inertial measurement units (IMUs) against ground reaction force detection. **TRD**: treadmill; **OVG**: overground; **nr**: not recorded.

Author (year)	Population	IMU Setup	Number of IMUs	Sample size	Walking condition	# Steps	Steps total	Error HS (ms)	Error TO (ms)
Hansen (2002) [35]	Healthy adults	Ankle	2	4	OVG	nr	nr	8	16
Zijlstra(2002) [27]	Healthy adults	Sacrum	1	15	TRD	20	300	nr	nr
Gonzales (2010) [34]	Healthy adults	Sacrum	1	11	OVG	156	1719	13	9
Bugané (2012) [33]	Healthy adults	Sacrum	1	22	OVG	50	1100	nr	nr
Boutaayamou (2015) [28]	Healthy adults	Heels and Toes	4	7	OVG	247	nr	7.2	11.8
Maqbool (2017) [29]	Amputees	Thigh	1	2	OVG	50	100	50	50
	Healthy adults	Thigh	1	8	OVG	50	400	11	8
Panebianco (2018) [30]	Healthy adults	Sacrum	5	35	OVG	24	420	70	66
	Healthy adults	Ankle	5	35	OVG	24	420	62	29
	Healthy adults	Foot	5	35	OVG	24	420	63	20
Strick (2024) [31]	Healthy adults	Thigh	1	19	OVG	43	830	77	16
Yang (2024) [32]	Healthy adults	Thigh	1	2	OVG	nr	nr	6.5	22
**Median**			**1**	**8**		**43**	**400**	**56**	**21**
**Mean**			**2**	**12.6**		**76.8**	**410.3**	**43.3**	**27.9**
**SD**			**1.5**	**11.7**		**91.4**	**266.8**	**30.1**	**20.1**

Supplementary Table 3. Studies validating gait event detection methods based on inertial measurement units (IMUs) against optical motion capture (OMC), and pressure-based techniques (foot switches, pressure mats and insoles. **SCI**: spinal cord injured patients; **OVG**: overground, **TRD**: treadmill; **nr**: not recorded.

Author (year)	Population	Ground truth	IMU Setup	# IMUs	Sample size	Floor	# Steps	Steps total	Error HS (ms)	Error TO (ms)
Jasiewicz (2006) [36]	Healthy adults	Footswitch	Foot dorso / Shank	4	19	OVG	51	978	11	19
	SCI	Footswitch	Foot dorso / Shank	4	13	OVG	90	1170	24	23
McCamley (2012) [37]	Healthy adults	Pressure mat	Sacrum	1	18	OVG	nr	nr	20	30
Godfrey (2014) [38]	Healthy adults	Pressure mat	Sacrum	1	24	OVG	5	nr	nr	nr
Sejdić, E. (2015) [39]	Older adults	OMC	Sacrum	1	35	TRD	nr	nr	nr	nr
Del Din, S. (2015) [40]	Older adults	Pressure mat	Sacrum	1	30	OVG	nr	nr	nr	nr
	Parkinson	Pressure mat	Sacrum	1	30	OVG	nr	nr	nr	nr
Boutaayamou (2015) [28]	Healthy adults	OMC	Foot (heel and dorso)	4	7	OVG	35	247	1.3	1.8
Romijnders (2021) [41]	Older adults	OMC	Shin	2	11	OVG	53	589	14	25
	Parkinson	OMC	Shin	2	14	OVG	42	589	26	25
	AVC	OMC	Shin	2	9	OVG	65	589	17	6
Nazarahari (2022) [42]	Healthy adults	Pressure insole	Foot	4	7	OVG	148	1039	20	20

Author (year)	Population	Ground truth	IMU Setup	# IMUs	Sample size	Floor	# Steps	Steps total	Error HS (ms)	Error TO (ms)
Voisard (2024) [43]	Healthy adults	OMC	Foot (dorso)	2	13	OVG	8	410	7	8
	Multiple sclerosys	OMC	Foot (dorso)	2	29	OVG	27	442	15	23
Brahimetaj (2024) [44]	Healthy adults	OMC	Sacrum	1	40	OVG	nr	nr	45	28
Bugané (2012) [33]	Healthy adults	OMC	Sacrum	1	22	OVG	50	1100	nr	nr
Digo (2020) [45]	Healthy elderly	OMC	Sacrum	1	14	OVG	30	420	nr	nr
	Healthy elderly	OMC	Shank / Ankle	4	14	OVG	30	420	nr	nr
Hundza (2014) [46]	Parkinson	OMC	Shank / Ankle	4	6	OVG	nr	750	nr	nr
	Healthy adults	OMC	Shank / Ankle	4	7	OVG	nr	875	nr	nr
Kluge (2017) [47]	Helathy elderly	OMC	Ankle	2	25	OVG	nr	1037	nr	nr
	Parkinson	OMC	Ankle	2	4	OVG	nr	129	nr	nr
Larsen (2024) [48]	Healthy adults	OMC	Thigh	1	52	TRD	733	38116	32	32
Lee (2009) [49]	Healthy adults	Footswitch	Ankle	2	19	OVG	20	380	32	nr
**Median**				**2**	**14.0**		**42.0**	**589**	**20**	**23**
**Mean**				**2.1**	**18.1**		**92.0**	**2738**	**20**	**20**
**SD**				**1.3**	**11.9**		**180.7**	**8834.8**	**11.7**	**9.8**
